# Complete Junctional Scotoma Secondary to Metastatic Melanoma: A Rapidly Progressive Presentation

**DOI:** 10.7759/cureus.97757

**Published:** 2025-11-25

**Authors:** Rochelle Boguslavskiy, Madison Tharp, Willy Gan, Marc A Swerdloff

**Affiliations:** 1 Neurology, Florida Atlantic University Charles E. Schmidt College of Medicine, Boca Raton, USA; 2 Neurology, Marcus Neuroscience Institute, Baptist Health Boca Raton Regional Hospital, Boca Raton, USA

**Keywords:** cavernous sinus lesions, cranial nerve involvement, junctional scotoma, meckels cave, metastatic melanoma, neurological exam, optic chiasm compression, trigeminal sensory loss, visual field defects

## Abstract

Junctional scotomas are distinctive visual field defects caused by lesions at the junction of the optic nerve and optic chiasm. The classic presentation includes ipsilateral vision loss with a contralateral superior temporal field deficit. A complete junctional scotoma involves more extensive damage, resulting in visual field loss across the entire contralateral temporal field. We present the case of a woman in her 30s with a recent diagnosis of metastatic melanoma who developed unilateral vision loss that progressed to a complete junctional scotoma within three days. Her symptoms were rapidly evolving and included trigeminal sensory changes and cranial nerve involvement. This case highlights the importance of visual field testing and thorough neurological examination in lesion localization. Although rare, metastatic tumors such as melanoma can produce aggressive neuro-ophthalmologic deficits, necessitating prompt recognition and intervention.

## Introduction

Lesions involving the optic chiasm typically produce bitemporal hemianopia due to the disruption of crossing nasal retinal fibers [1]. In contrast, junctional scotomas result from a combined compression of the ipsilateral optic nerve and the adjacent portion of the optic chiasm. The classic presentation includes ipsilateral vision loss with a contralateral superior temporal visual field deficit, reflecting involvement of the contralateral inferior nasal fibers. When both inferior and superior nasal fibers are affected, a complete junctional scotoma occurs, characterized by visual field loss across the entire contralateral temporal visual field and more profound visual impairment [2].

Compression at the optic nerve-chiasm junction may arise from various etiologies, including aneurysms, arteriovenous malformations, trauma, demyelinating diseases such as multiple sclerosis, and intracranial masses. While pituitary adenomas and meningiomas are common causes, metastatic tumors are less frequent but often present with more aggressive and rapidly progressive symptoms [3].

Visual field testing remains a critical tool for lesion localization and monitoring. Although pituitary adenomas typically cause gradual visual decline due to their slow growth, metastatic lesions such as melanoma may lead to rapid deterioration. We present the case of a woman in her 30s with a recent diagnosis of metastatic melanoma who developed unilateral vision loss that progressed to a complete junctional scotoma within three days.

## Case presentation

A woman in her 30s with a history of migraines presented to the Emergency Department with a three-week history of worsening headache. Initially rated as 2/10 in intensity, the pain escalated to 10/10 shortly before arrival. She described the headache as a right-sided, shock-like sensation occurring every five to 10 minutes, distinct from her typical migraines, which are throbbing and constant in nature. The pain disrupted sleep and worsened with forward flexion. Associated symptoms included right-sided facial numbness around the right eye radiating to the occiput, nausea, and blurry vision in the right eye.

**Figure 1:**
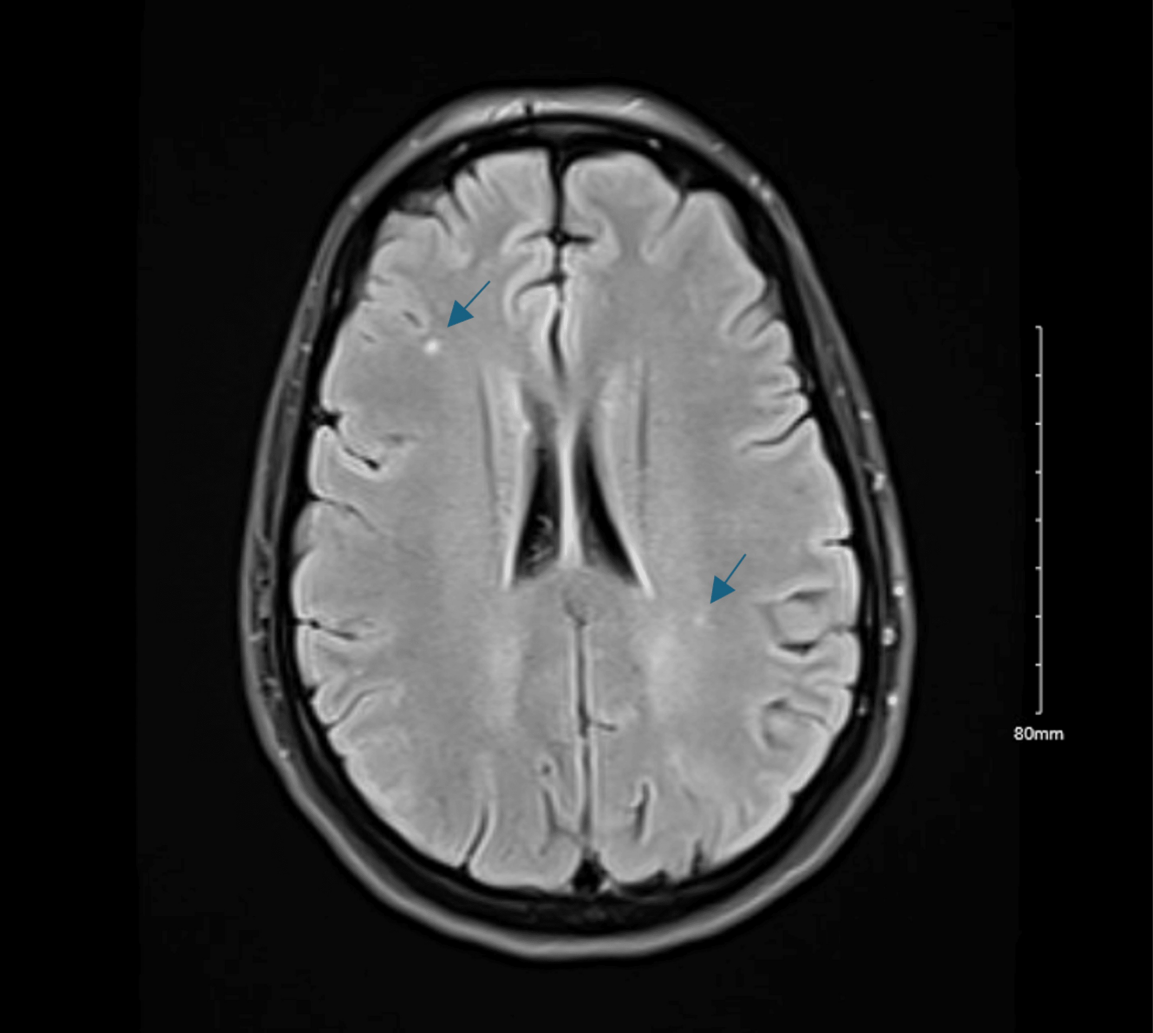
MRI of the Brain Revealing Migraine-Associated White Matter Changes Axial MRI of the brain without contrast demonstrating tiny, nonspecific T2/FLAIR hyperintense foci within the deep white matter of the right frontal and left parietal lobes. These findings are consistent with migraine-related changes. MRI: magnetic resonance imaging; FLAIR: Fluid-attenuated inversion recovery.

Neurological examination on day 1 revealed full visual fields but diminished facial sensation in the right V1-V2 distribution. MRI of the brain without contrast showed small T2/FLAIR (fluid-attenuated inversion recovery) hyperintensities in the frontal and parietal lobes, consistent with migraine-related changes (Figure 1). Her headache resolved after administration of a migraine cocktail on hospital day 2, though facial numbness and blurry vision persisted. Magnetic resonance (MR) venography showed patent dural venous sinuses. Ophthalmologic evaluation revealed visual acuity of 20/40 bilaterally, reactive pupils, and no afferent pupillary defect.

**Figure 2:**
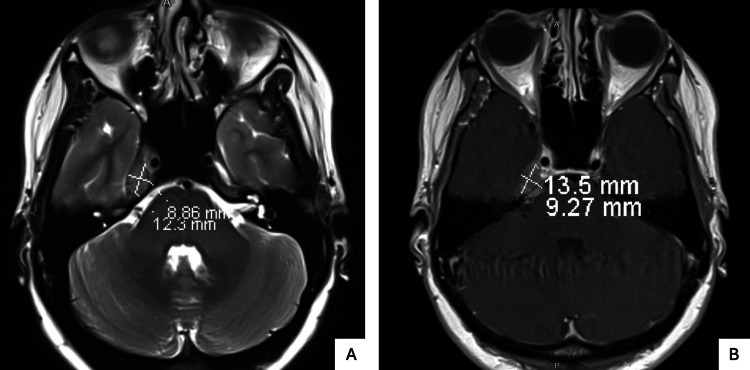
Contrast-Enhanced MRI of the Brain and IACs Revealing a Lesion Encasing the Right Trigeminal Nerve Extending into Meckel’s Cave MRI of the brain and IACs, with and without contrast, demonstrating a well-defined mass measuring approximately 13×9 mm. The lesion partially encases the cisternal segment of the right trigeminal nerve and extends into Meckel’s cave. Panel A shows the mass on axial T2-weighted imaging; Panel B shows post-contrast enhancement on axial T1-weighted imaging. IACs: Internal auditory canals.

Due to persistent sensory symptoms, MRI of the brain and internal auditory canals with contrast was performed on day 3, revealing a well-defined mass partially encasing the cisternal segment of the right trigeminal nerve and extending into Meckel’s cave (Figure 2). CT imaging of the chest, abdomen, and pelvis was unremarkable. Neurosurgery recommended outpatient follow-up, and the patient was discharged on gabapentin and sumatriptan.

Following discharge, she had an extensive workup, including a biopsy, and was diagnosed with metastatic melanoma of unknown primary (MUP) involving the brain and lungs. She underwent two hemicranial resections with stereotactic radiosurgery at Meckel's cave and initiated immunotherapy with nivolumab and relatlimab (Opdualag). One year later, she returned with a two-day history of worsening headache and new peripheral visual shadows in the right eye. The headache was throbbing and pressure-like, relieved by topiramate and acetaminophen. She was also diagnosed with hypopituitarism secondary to mass effect and started on hydrocortisone.

On day 1 of her second admission, her right pupil was ovoid with absent direct and consensual light reflexes. The right corneal reflex was absent. She exhibited a mild right abducens nerve palsy with limited lateral gaze and rightward gaze evoked paretic nystagmus along with a right oculomotor nerve palsy with more pronounced limitation in upward gaze. A reverse Marcus-Gunn pupil was present in her left eye with indirect dilation of her right eye. Visual acuity was reduced to no light perception in the right eye and 20/40 in the left. Facial hypoesthesia persisted in the right V1-V2 distribution, but had progressed to involve V3.

**Figure 3:**
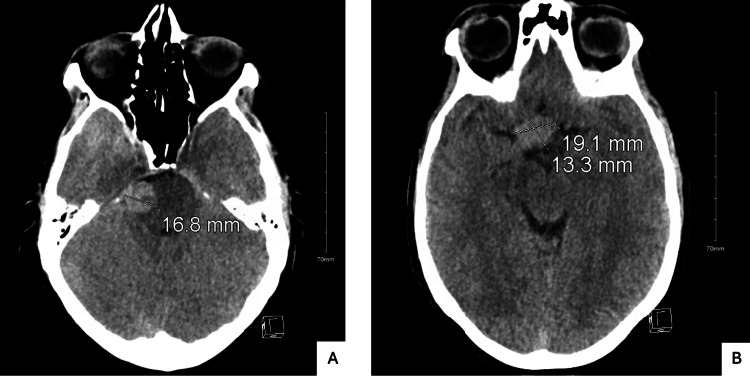
Non-Contrast CT of the Brain Revealing a New Pontine and Suprasellar Mass Non-contrast CT of the brain obtained one year later. Panel A shows a new mass adjacent to the right pons measuring 16.8 mm. Panel B reveals a suprasellar mass measuring 19.1×13.3 mm, which was not present on prior imaging. CT: computed tomography.

**Figure 4:**
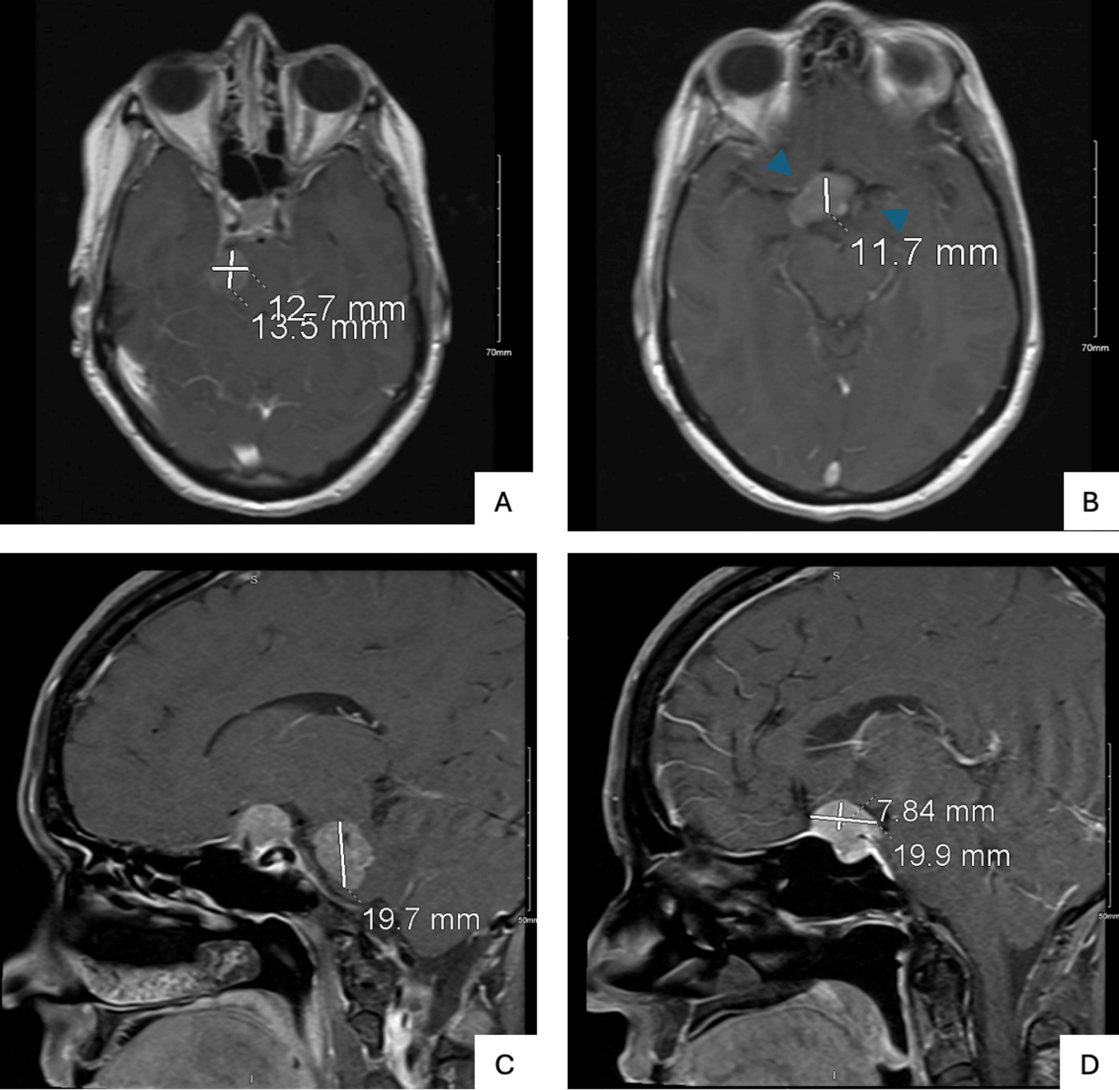
Post-Contrast MRI Brain and Pituitary Revealing a Suprasellar Mass with Optic Chiasm Displacement MRI of the brain and pituitary, with and without contrast, obtained one year later. Axial T1-weighted post-contrast images (A, B) and sagittal T1-weighted post-contrast images (C, D) demonstrate a well-defined suprasellar mass with contrast enhancement measuring approximately 8x20 mm. The lesion compresses and displaces the optic chiasm and partially encases the bilateral cavernous internal carotid arteries (ICAs) as indicated by blue arrowheads.

CT of the brain showed a new suprasellar mass measuring approximately 19×13 mm and a lesion adjacent to the right pons measuring approximately 17 mm (Figure 3). The previously visualized Meckel’s cave mass was not well seen. MRI of the brain and pituitary with contrast demonstrated a well-defined, enhancing suprasellar mass compressing and displacing the optic chiasm, with partial encasement of the bilateral cavernous internal carotid arteries (Figure 4). MRI of the orbits showed no intraorbital mass or infiltrative process.

**Figure 5:**
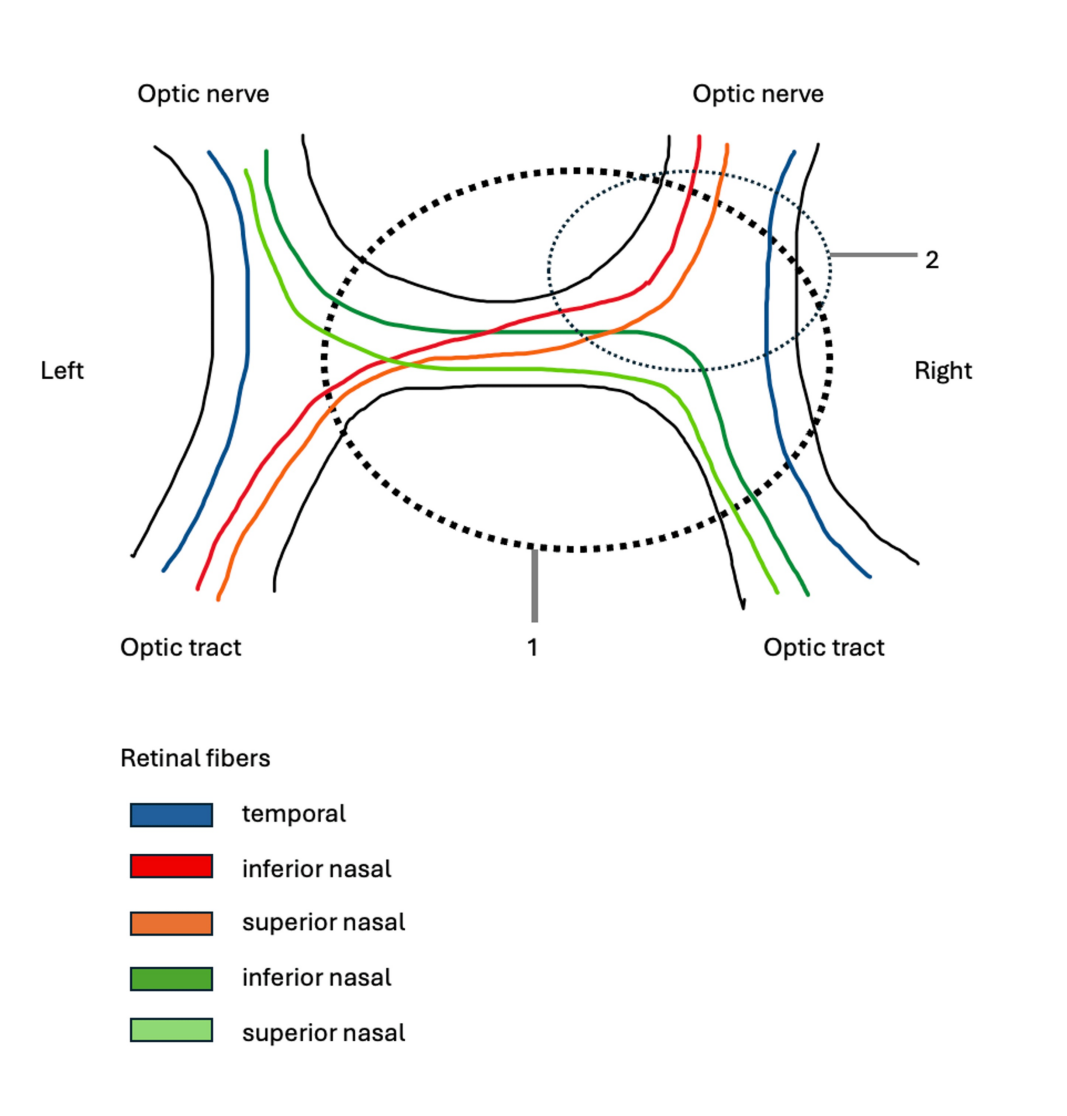
Anatomy of the Visual Pathway at the Optic Chiasm Illustrating a Classic and Complete Junctional Scotoma Diagram of the optic chiasm illustrating the anatomical basis of junctional scotomas. A dotted circle highlights the location of a lesion that would result in (1) a complete junctional scotoma - caused by compression of both superior and inferior nasal fibers of the contralateral optic nerve - and (2) a classic junctional scotoma - resulting from selective compression of inferior nasal fibers. The visual pathway is labeled to show the crossing of nasal retinal fibers and the ipsilateral course of temporal retinal fibers. Image credit: Dr. Rochelle Boguslavskiy.

**Figure 6:**
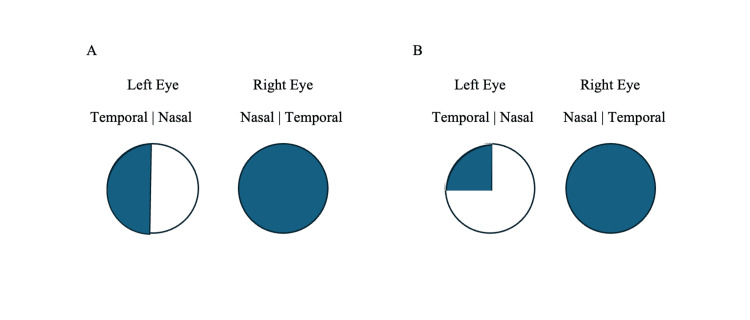
Visual Field Findings for a Complete and Classic Junctional Scotoma Illustration of the visual field deficits found in complete and classic junctional scotomas. Panel A shows our patient's complete junctional scotoma on visual field testing with upper and lower temporal field deficits in the left eye and complete visual field loss in the right eye. Panel B shows an example of a classic junctional scotoma on visual field testing with a superior temporal field deficit in the left eye and complete visual field loss in the right eye.

On day 3 of her second admission, the patient reported sudden vision loss in the left eye. Examination revealed absent direct and consensual pupillary light reflexes in the left eye. Visual field testing demonstrated deficits in both upper and lower temporal fields of the left eye. Given her prior biopsy-confirmed results, these findings were most consistent with a complete junctional scotoma, secondary to metastatic melanoma compressing the optic chiasm and adjacent right optic nerve (Figures 5, 6). She was transferred to a tertiary care center for further neurosurgical evaluation where she underwent suprasellar tumor debulking and decompression.

Following surgery, the patient reported improved vision in her left eye with no recovery noted in her right eye. Postoperative pathology confirmed metastatic melanoma. She initiated stereotactic radiation targeting both the previously irradiated Meckel's cave lesion and the newly identified suprasellar mass. Chemotherapy with temozolamide was started and she continued treatment with Opdualag. She was subsequently diagnosed with tumor-associated pituitary failure and was closely followed by endocrinology. Later, dexamethasone was initiated due to worsening left eye vision and new facial swelling. Despite aggressive multimodal therapy, her metastatic melanoma continued to progress. She was ultimately transitioned to hospice care, where she passed away (Table 1).

**Table 1:** Timeline Summary of Patient Presentation, Diagnostic Findings, and Interventions This table provides a summary of the patient's presentation including symptoms, neurological findings, imaging results, and key interventions across two hospital admissions and the post-discharge period. The chronological order highlights the progression from initial headache with visual and sensory changes to the development of a complete junctional scotoma. FLAIR: Fluid-attenuated inversion recovery.

Day/Timeframe	Symptoms/Findings	Neurological examination	Imaging Results	Interventions
3 weeks prior to first admission	Worsening right-sided headache 2/10-->10/10 shock-like, sleep disrupting, right sided facial numbness, nausea, and right eye blurry vision	-	-	-
Day 1 (firstadmission)	Persistent headache, right-sided facial numbness, right eye blurry vision	Full visual fields, diminished facial sensation in right V1-V2 distribution	MRI brain with no contrast (Figure 1): T2/FLAIR hyperintensities in frontal/parietal lobes (migraine-related)	-
Day 2	No headache; persistent sensory symptoms and blurry vision	Visual acuity 20/40 bilaterally, diminished facial sensation in right V1-V2 distribution, reactive pupils, no afferent pupillary defect	MR venography: patent dural venous sinuses	Migraine cocktail
Day 3	Persistent facial numbness	Diminished facial sensation in right V1-V2 distribution	MRI of the brain and internal auditory canals with contrast (Figure 2): well-defined mass partially encasing the cisternal segment of the right trigeminal nerve and extending into Meckel’s cave; CT Chest, Abdomen and Pelvis with contrast: unremarkable	Discharged on gabapentin and sumatriptan with neurosurgery follow-up
Post-discharge after first admission	-	-	Extensive workup: biopsy confirmed metastatic melanoma to brain and lungs	Two hemicranial resections, stereotactic radiation, and immunotherapy (nivolumab+relatlimab)
1 year later (secondadmission)	Two days of worsening throbbing, pressure like headache with peripheral visual shadows in right eye; diagnosed with hypopituitarism	Multiple cranial nerve palsies, no light perception in right eye, 20/40 visual acuity in left eye, persistent facial hypoesthesia in right V1-V2 distribution progressing to involve V3	CT brain without contrast (Figure 3): new suprasellar mass measuring approximately 19×13 mm and a lesion adjacent to the right pons measuring approximately 17 mm; MRI brain and pituitary with contrast (Figure 4): well-defined, enhancing suprasellar mass compressing and displacing the optic chiasm, with partial encasement of the bilateral cavernous internal carotid arteries; MRI orbits with contrast: no intraorbital mass or infiltrative process.	Topiramate, acetaminophen, and hydrocortisone
Day 3 (secondadmission)	Sudden vision loss in left eye	Absent pupillary light reflexes in left eye. Visual field testing with deficits in temporal fields of left eye.	-	Transferred for neurosurgical evaluation
Post-discharge after second admission	Improved vision in left eye post-surgery; diagnosed with tumor associated pituitary failure; had worsening left eye vision and new facial swelling	-	-	Suprasellar tumor debulking and decompression followed by stereotactic radiation, chemotherapy and immunotherapy with temazolamide and Opdualag, and dexamethasone; later transitioned to hospice care

## Discussion

This case illustrates a rare and rapidly progressive development of a complete junctional scotoma secondary to metastatic melanoma. The patient initially presented with an atypical headache and right-sided facial pain, later attributed to a well-defined mass in Meckel’s cave. Her symptoms, including shock-like sensations, shooting pain, and numbness in the V1-V2 distribution that progressed to involve V3, accompanied by the finding of an absent right corneal reflex, were consistent with trigeminal nerve involvement at Meckel’s cave.

Tumors within Meckel’s cave are uncommon, with schwannomas and meningiomas being the most frequently reported [4]. Patients with benign lesions are less likely to experience pain or paresthesias in the trigeminal distribution compared to those with metastatic involvement. However, this difference is largely influenced by the extent of trigeminal nerve involvement [5]. This distinction underscores the importance of considering metastatic disease in patients presenting with acute and progressive trigeminal sensory changes.

Following her diagnosis of metastatic melanoma, the patient underwent surgical resection and immunotherapy with nivolumab and relatlimab (Opdualag). Despite treatment, she returned one year later with complete vision loss in the right eye, consistent with ipsilateral optic nerve compression [6]. Examination revealed an ovoid, amaurotic right pupil with absent direct and consensual light reflexes, confirming significant optic nerve dysfunction. Within three days, she developed visual field deficits in the upper and lower temporal quadrants of the left eye, along with loss of pupillary reflexes, indicating tumor progression to the junction of the optic chiasm [7].

Her visual field loss was consistent with a complete junctional scotoma, involving compression of both inferior and superior nasal fibers of the contralateral optic nerve [2,7]. This pattern spares only the contralateral nasal field and results in profound visual impairment. Barton and Özturan’s review of 17 cases emphasized that such complete defects were more commonly identified prior to the widespread use of advanced imaging, highlighting the enduring value of detailed neuro-ophthalmological examination in lesion localization [2].

Visual field testing remains a critical tool for monitoring disease progression and guiding treatment [8]. In this case, it helped identify the lesion at the junction of the optic chiasm and optic nerve with radiographic confirmation of a suprasellar mass. The patient also exhibited mild right abducens and third nerve palsies, consistent with cavernous sinus involvement, as well as persistent hypoalgesia in the V1-V3 distribution due to the proximity of the cavernous sinus to Meckel’s cave [9,10].

Although imaging confirmed the suprasellar mass compressing the optic chiasm, the diagnosis of a complete junctional scotoma relied heavily on clinical findings. The combination of visual field testing and serial neurological examinations provided critical insight into the rapid progression of her metastatic disease.

This case highlights the aggressive nature of metastatic melanoma and its potential to cause rapidly evolving neuro-ophthalmologic deficits. While rare, metastatic tumors should be considered in patients presenting with acute trigeminal sensory changes and progressive visual loss. Early recognition through a thorough neurological examination and visual field testing is essential for timely diagnosis and intervention. Given the rarity of this presentation, data on prognostic implications, long-term outcomes, and systemic therapies in similar cases are limited and represent important areas for future investigation.

## Conclusions

This case underscores the importance of maintaining a broad differential diagnosis when evaluating patients with rapidly progressive visual loss and trigeminal sensory changes. Metastatic melanoma, though rare in this context, can present with aggressive neuro-ophthalmologic deficits, including complete junctional scotoma. Metastatic lesions in Meckel’s cave are uncommon, but they should be considered in patients with acute, evolving trigeminal nerve pain. Visual field testing and thorough neurological examination remain essential tools for early lesion localization and clinical decision-making. Notably, clinical findings may precede radiographic changes, reinforcing the value of bedside assessment in guiding timely diagnosis and intervention. Long-term outcomes and treatment efficacy in similar cases remain important areas for future investigation. 
